# Genetic Basis of Emerging Vancomycin, Linezolid, and Daptomycin Heteroresistance in a Case of Persistent Enterococcus faecium Bacteremia

**DOI:** 10.1128/AAC.02007-17

**Published:** 2018-03-27

**Authors:** Kieran I. Chacko, Mitchell J. Sullivan, Colleen Beckford, Deena R. Altman, Brianne Ciferri, Theodore R. Pak, Robert Sebra, Andrew Kasarskis, Camille L. Hamula, Harm van Bakel

**Affiliations:** a Icahn Institute for Genomics and Multiscale Biology, Department of Genetics and Genomic Sciences, Icahn School of Medicine at Mount Sinai, New York, New York, USA; b Division of Infectious Diseases, Department of Medicine, Icahn School of Medicine at Mount Sinai, New York, New York, USA; c Department of Pathology, Icahn School of Medicine at Mount Sinai, New York, New York, USA

**Keywords:** E. faecium, VRE, daptomycin, *fabF*, heteroresistance, linezolid, vancomycin

## Abstract

Whole-genome sequencing was used to examine a persistent Enterococcus faecium bacteremia that acquired heteroresistance to three antibiotics in response to prolonged multidrug therapy. A comparison of the complete genomes before and after each change revealed the emergence of known resistance determinants for vancomycin and linezolid and suggested that a novel mutation in *fabF*, encoding a fatty acid synthase, was responsible for daptomycin nonsusceptibility. Plasmid recombination contributed to the progressive loss of vancomycin resistance after withdrawal of the drug.

## TEXT

Multidrug-resistant (MDR) Enterococcus faecium is a common cause of nosocomial infections (1). Resistance to ampicillin or vancomycin occurs in approximately 90% and 80% of nosocomial E. faecium in the United States, respectively (1). Linezolid and daptomycin are currently used as first-line treatment options for vancomycin-resistant E. faecium (VREfm) (2, 3). Although resistance to both agents remains rare among enterococci (<1% for linezolid and <2% for daptomycin) (4, 5), the emergence of resistance during treatment with each drug has been documented in multiple cases (5–7) and can pose significant challenges for infection management. A complication in assessing emerging resistance during infection is that bacterial isolates sometimes show a range of susceptibilities to a particular antibiotic due to genetic, epigenetic, or nongenetic heterogeneity within the isolates; a phenomenon known as “heteroresistance” (8, 9). Heteroresistance can cause significant diagnostic and therapeutic complications, and it has been associated with persistent infections and increased mortality rates (10–13). Nevertheless, the full extent of heteroresistance and its broader clinical relevance remain unclear, and only a few cases have been reported in E. faecium (14–16). In this report, we used complete genome sequencing to characterize the genetic changes underlying emerging vancomycin, linezolid, and daptomycin heteroresistance in a case of persistent E. faecium infection. Note that we will refer to daptomycin resistance throughout for consistency, rather than the accepted term “nonsusceptibility.”

**FIG 1:**
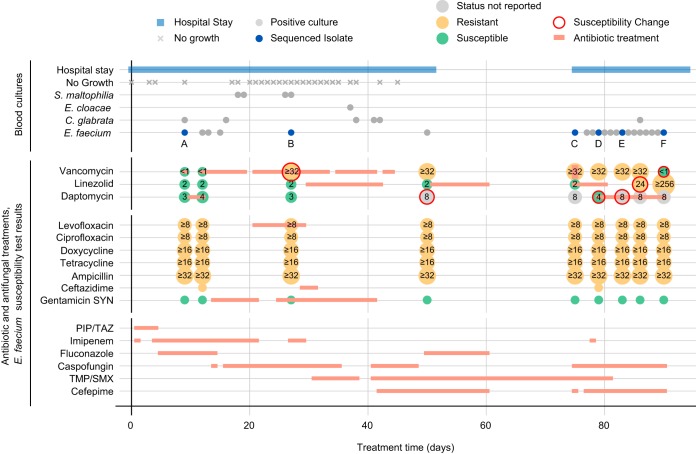
Timeline of antimicrobial treatments and susceptibility changes. Hospital stays and blood culture test results (top) are shown together with antibiotic and antifungal treatment regimes and E. faecium susceptibility profiles (bottom). Positive blood cultures are grouped by pathogen, and the E. faecium isolates selected for sequencing are highlighted in blue and labeled A to F. Automated broth microdilution test results (VITEK2) are shown as colored circles containing MIC values (μg/ml), where available. Red circle outlines indicate instances of acquired or lost resistance. See legend for further details.

In 2015, a 65-year-old male was admitted to Mount Sinai Hospital (MSH) who developed an E. faecium bacteremia that spanned 3 months and two hospital stays (Fig. 1). E. faecium was detected in 21 of the 48 blood samples collected from the patient, with additional sporadic detections of Candida glabrata, Stenotrophomonas maltophilia, or Enterobacter cloacae. Automated broth microdilution testing (VITEK2) of single colonies from each isolate culture showed that E. faecium acquired resistance to vancomycin (day 27), daptomycin (day 50), and linezolid (day 86) following treatment with each agent (Fig. 1). After the cessation of vancomycin therapy, vancomycin susceptibility was restored on day 90.

To characterize the susceptibility changes in more detail, we collected six isolates from the original blood cultures before and after each change (Fig. 1, labeled A to F). There were no differences in E. faecium colony morphology within or between isolates. For each isolate, we tested four isogenic strains derived from single colonies, in duplicates, to confirm vancomycin, linezolid, and daptomycin susceptibilities (Table 1). The vancomycin Etest results were consistent with the VITEK2 reports for all isolates except C and E, where susceptibility was restored in two of four strains tested. The mixed susceptibility phenotypes suggested the presence of a vancomycin heteroresistant population; therefore, we performed additional Etests on the original blood isolates, which revealed sparsely distributed isolated colonies within the zones of inhibition for isolates C, E, and F. Isolate A was uniformly susceptible, while B and D were uniformly resistant. The lack of heteroresistance in B and D may be a result of selection pressure due to vancomycin treatment (Fig. 1; see also supplemental material) prior to or at the time of their collection. The linezolid Etests confirmed resistance for isolate F, albeit at lower MICs, and further identified resistance in isolates D and E, for which results had not been reported by the automated broth microdilution. Notably, there was significant variation in MICs among the resistant strains tested for isolates D to F, ranging from 24 to 96 μg/ml. Daptomycin Sensititre assays also showed variability within and between samples. Isolate C tested consistently at the nonsusceptibility threshold MIC of 4 μg/ml, compared to 8 μg/ml by VITEK2, whereas isolates D to F yielded mixed results with MICs ranging from 4 to ≥8 μg/ml. Taken together, our results are consistent with the emergence of an E. faecium population with heteroresistance to vancomycin, linezolid, and daptomycin.

**TABLE 1:** Vancomycin, linezolid, and daptomycin susceptibilities of patient isolate clones

Agent	Isolate	Day	Susceptibility and MIC (μg/ml) from:^*a*^				
			Clinical test (VITEK2)^*b*^	Confirmation test			
				1^*c*^	2	3	4
Vancomycin^*d*^	A	9	S (<0.5)	S (<1)	S (<1)	S (<1)	S (<1)
	B	27	R (≥32)	R (≥256)	R (≥256)	R (≥256)	R (≥256)
	C	75	R (≥32)	R (≥256)	S (<1)	S (<1)	R (≥256)
	D	79	R (≥32)	R (≥256)	R (≥256)	R (≥256)	R (≥256)
	E	83	R (≥32)	S (<1)	R (≥256)	S (<1)	R (≥256)
	F	90	S (<0.5)	S (<1)	S (<1)	S (<1)	S (<1)
Linezolid^*d*^	A	9	S (2)	S (2)	S (2)	S (2)	S (2)
	B	27	S (2)	S (2)	S (2)	S (2)	S (2)
	C	75	S (2)	S (2)	S (2)	S (2)	S (2)
	D	79	—	R (48)	R (24)	R (24)	R (32)
	E	83	—	R (96)	R (48)	R (64)	R (64)
	F	90	R (≥256)	R (64)	R (64)	R (24)	R (96)
Daptomycin^*e*^	A	9	S (3)	S (2)	S (2)	S (2)	S (2)
	B	27	S (3)	S (2)	S (2)	S (2)	S (2)
	C	75	— (8)	S (4)	S (4)	S (4)	S (4)
	D	79	S (4)	R (≥8)	R (≥8)	S (4)	R (≥8)
	E	83	— (8)	R (≥8)	R (≥8)	R (≥8)	S (4)
	F	90	— (8)	R (≥8)	R (≥8)	R (≥8)	S (4)

a R, resistant; S, susceptible; —, not reported.

b Maximum tested MIC for vancomycin by automated broth microdilution was 32 μg/ml.

c Strain set selected for complete genome sequencing.

d MICs determined by Etest assays performed in duplicates.

e MICs determined by Sensititre assays performed in duplicates.

**FIG 2:**
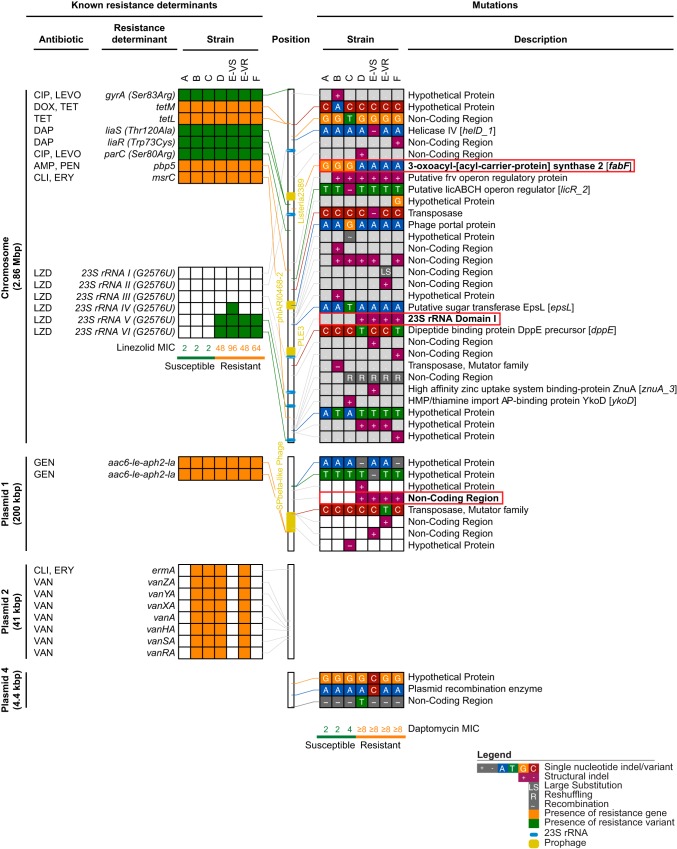
Resistance determinants and mutations identified in patient strains. Known antibiotic resistance determinants (left) are shown together with all other mutations (right) identified between strains. Connecting lines indicate the location of each determinant or mutation in the strain A genome (center). Locations of prophage insertions and 23S rRNA loci are indicated. Mutations matching the daptomycin susceptibility patterns are boxed in red and highlighted in bold. See legend for further details. CIP, ciprofloxacin; LEVO, levofloxacin; DOX, doxycycline; TET, tetracycline; DAP, daptomycin; AMP, ampicillin; PEN, penicillin; CLI, clindamycin; ERY, erythromycin; LZD, linezolid; GEN, gentamicin; VAN, vancomycin.

We next performed whole-genome sequencing for strain 1 of each of the six isolates (Table 1) and an additional vancomycin resistant strain from the heteroresistant E isolate (E-VR). Complete genomes were obtained for each strain using PacBio single molecule real-time long-read sequencing and Illumina short-read sequencing. Multilocus sequence typing indicated that all strains were sequence type 736 (ST736). This clone is associated with reduced susceptibility and nonsusceptibility to daptomycin (MIC = 3 to 4 μg/ml) due to the presence of *liaS* Thr120→Ala and *liaR* Trp73→Cys substitutions (17). The same substitutions were identified in all sequenced strains (Fig. 2) and presumably explain the low-basal-level daptomycin tolerance exhibited by strains A to C (Table 1) (18, 19). The genomes for each strain were nearly identical, with a maximum of seven single nucleotide variants (SNVs) separating any two genomes (see Table S1). Thus, a single clone of E. faecium was responsible for the infection, and the resistance emerged as a result of genetic changes within this clone. A further comparison of our strain genomes to 27 ST736 E. faecium genomes deposited in GenBank (see Fig. S1) yielded a maximum distance of 88 core genome SNVs between strain A and a 2012 VREfm isolate from Washington, USA. Notably, most ST736 genomes were derived from clinical isolates collected in the New York metropolitan (NYC) area (hospital A, B, and MSH). The small genetic distance between the ST736 genomes relative to the reported mutation rate for VREfm strains of 9.4E−6 substitutions per nucleotide per year (20) suggests that the infection in our patient was part of a larger clonal spread of E. faecium ST736 in the NYC region in recent years.

We further examined the genomes for known antibiotic resistance determinants (Fig. 2, left) that matched the susceptibility changes. Each strain contained a 2.86-Mb chromosome and three plasmids of approximately 200 kb (p1), 10 kb (p3), and 4 kb (p4) in size (Table S1 and Fig. S2). An additional 41-kb plasmid (p2) carrying the *vanA* operon in a 11,654-bp BC1 Tn*1546*-type transposon (21, 22) was identified in vancomycin-resistant strains only (Fig. 2; see also Table S1 and Fig. S2), explaining the observed susceptibility profiles for this antibiotic. One strain, E-VS, contained an 80-kb plasmid (p5) that was not found in any other strain. The emerging linezolid resistance was accounted for by a G2,576U substitution (23) present in two or three of the six copies of the 23S rRNA in strains D, E-VS, E-VR, and F (Fig. 2). We did not find other determinants associated with linezolid resistance, such as other mutations in the 23S rRNA gene (23–25) or genes encoding ribosomal proteins L3, L4, and L22 (23, 26) or the acquisition of *cfr* (27, 28) or *optrA* (29). Consistent with previous observations (24), strains with higher linezolid MICs had more G2,576U mutant 23S rRNA gene copies, indicating that these mutations were responsible for the observed heteroresistance phenotype. Interestingly, we observed consistently higher MICs per 23S rRNA mutant copy than a previous gene dosage study, which reported a maximum MIC of 32 μg/ml for 2 to 3 mutant 23S rRNA copies (24) versus 48 to 96 μg/ml in our strains. This difference may be due to the presence of a 1,417-bp IS*Enfa3*-like mobile element within domain I of 23S rRNA gene copy III in all four resistant strains (Fig. 2), as disruption of one of the wild-type rRNA genes likely increased the effective dosage of mutant 23S rRNAs.

Daptomycin resistance (≥8 μg/ml MIC) arose concurrently with linezolid resistance in strains D to F. This increase in the MIC could not be explained by known daptomycin resistance determinants (18, 19, 30), indicating that a novel resistance mutation was present in these strains. It is unlikely that the mutations in 23S rRNA genes affected resistance, as daptomycin has been shown to remain effective in linezolid-resistant Enterococci and Staphylococci harboring the G2,576U substitution (31). Moreover, daptomycin MICs were not affected by differences in the numbers of mutant rRNA copies in our strains. Longitudinal genome comparisons between all strains identified 37 additional mutations (Fig. 2; Table S2). Only two other mutations occurred in a pattern consistent with the increase in daptomycin MIC: a nonsynonymous G962A mutation in *fabF* and an insertion of an IS*256*-like element in plasmid 1. The IS*256*-like element was inserted into a noncoding region between two carbohydrate metabolism genes encoding a putative fructokinase and a subunit of a sugar phosphotransferase system. The G962A mutation in *fabF* resulted in a Gly321→Asp change in the C-terminal domain of the β-ketoacyl-acyl carrier protein synthase it encodes. FabF is involved in membrane fatty acid metabolism (32, 33), consistent with the mechanism of action of daptomycin as a lipopeptide that disrupts the bacterial membrane. Notably, while FabF was not previously associated with daptomycin resistance in enterococci, a Pro137→Leu mutation in its ortholog was implicated in daptomycin resistance in Staphylococcus aureus (34). Taken together, these data suggest that the *fabF* mutation was responsible for the increased daptomycin MIC, although further studies are needed to confirm these findings and determine the mechanistic basis for its role in resistance.

**FIG 3:**
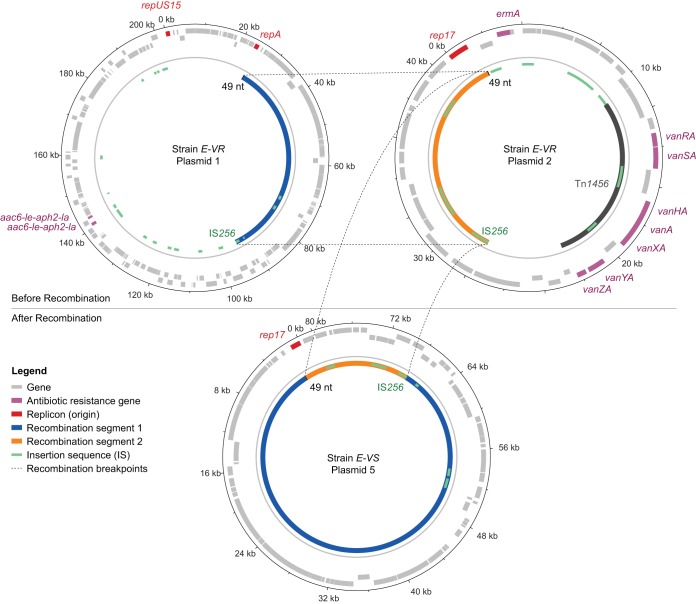
Excision of the *vanA* operon by plasmid recombination. Schematic representing recombination events between the 41-kb and 200-kb plasmids. Recombination was driven by the presence of the IS*256* insertion sequence and 49-nt sequence flanking each end of the conserved 69-kb and 14-kb regions of plasmids 1 and 2 from patient strain E-VR, respectively. The *vanA* operon was excised following recombination, resulting in loss of vancomycin resistance in strain E-VS.

Vancomycin heteroresistance in blood isolate E emerged 12 days after vancomycin treatment was stopped and within 4 days of that in the uniformly resistant D isolate (Fig. 1), prompting us to further examine the mechanism behind the loss of resistance. A comparison of vancomycin-susceptible (E-VS) and -resistant (E-VR) strains obtained from the same patient isolate revealed a recombination event between the 41-kb plasmid containing the *vanA* resistance gene cluster (p2) and the larger 200-kb plasmid (p1) present in all strains (Fig. 3). This resulted in a new 80-kb plasmid (p5) in strain E-VS that consisted of a 14-kb fragment of p2 and a copy of a 69-kb segment of p1. Notably, E-VS also retained an intact copy of p1, suggesting that the recombination event occurred during or shortly after p1 replication. The p5 plasmid retained the *rep17* replicon and *ori* from p2, whereas the excised 30-kb p2 fragment containing the *vanA* resistance operon was lost. Further sequence analysis identified homologous regions flanking the recombination sites in p1 and p2, consisting of a 1.3-kb insertion sequence (IS) IS*256* on one end and a 49-bp sequence on the other end. The 49-bp conserved sequence in p1 was directly adjacent to the *rep17* replicon. Thus, the p5 E-VS plasmid resulted from two separate homologous recombination events between p1 and p2 or from a combination of homologous recombination and IS*256* transposition (35).

The final vancomycin-susceptible strain from heteroresistant isolate F did not carry the 41-kb p2 plasmid or the p5 recombination product that we identified in E-VS. This suggests a continued loss of p2 elements after treatment ceased, as also evidenced by the smaller fraction of resistant colonies we observed in isolate F than in isolate E. The most likely explanation for this loss is that the presence of the vancomycin resistance plasmid reduces fitness. Indeed, a similar loss of *vanA* after withdrawal of vancomycin therapy has been described (14), and *in vitro* studies have shown that carriage of plasmid-mediated resistance incurs fitness costs (36). Nonetheless, a single dose of vancomycin on day 75 was sufficient to uniformly restore resistance in isolate D on day 79, demonstrating that the presence of even a small fraction of resistant bacteria enables rapid adaptation to treatment. Although we did not detect heteroresistance in the original A isolate, it is likely that low-level resistance was already present below the detectable limit. We consider horizontal gene transfer less plausible, especially considering that older ST736 isolates collected at Mount Sinai Hospital (37) all contained nearly identical 41-kb *vanA* resistance plasmids.

The present study demonstrates how E. faecium can evolve resistance to antibiotics of last resort through a combination of known and novel genetic mutations, which can ultimately result in treatment failure. Notably, heteroresistance emerged to three different antibiotics, underscoring its clinical relevance during persistent infections and suggesting that it may be more widespread than currently recognized. The clonal spread of ST736 in the NYC region is concerning, as its baseline reduced susceptibility to daptomycin may facilitate the selection of additional resistance variants and reduce the time to emergence of resistance during treatment. As such, the continued dissemination of ST736 and other clones carrying *liaFSR* mutations should be closely monitored. Notably, it has been shown that the addition of ampicillin can restore daptomycin bactericidal activity when *liaFSR* mutations are present (19, 38, 39), and combination therapy may help curtail further spread.

There are some limitations to our study. We did not sequence all colonies, and we may not have captured the full extent of genetic heterogeneity within the bacterial isolates. It is possible that mutations in other genes such as *gdpD* and *cls* (18, 19, 30) contributed to daptomycin heteroresistance. Our analyses were also limited to blood isolates, and a primary infection, such as the liver abscess the patient was originally diagnosed with, may have acted as a repository that was less accessible to antibiotics and from which bacteria continued to spread through the bloodstream. This does not detract from our main conclusions and would only serve to demonstrate the contribution of additional intrahost variability.

In summary, the ineffectiveness of commonly used agents, such as ampicillin and vancomycin, has increased our reliance on and use of last-line and off-label agents. The E. faecium genome has been shown to be highly adaptable, acquiring genes and chromosomal mutations that confer resistance to these last-line agents. We have demonstrated the applicability of complete-genome sequencing of longitudinal samples to comprehensively map all genomic changes responsible for acquired antibiotic resistance. With the increased reliance on newer agents in treating MDR E. faecium infections, there is a need to capture these events on a larger scale to better understand the underlying mechanisms responsible for acquired resistance of commonly used agents and their impact on treatment outcome.

### Accession number(s).

Genome sequences have been deposited in GenBank under BioProject accession number PRJNA407447.

## Supplementary Material

Supplemental material
