# Supplementary material for: Genetic Basis of Emerging Vancomycin, Linezolid, and Daptomycin Heteroresistance in a Case of Persistent Enterococcus faecium Bacteremia
Source: Antimicrob Agents Chemother. 2018 Mar 27;62(4):e02007-17. doi: 10.1128/AAC.02007-17 (PMC5913925; doi:10.1128/AAC.02007-17)
Supplement: Supplemental material [file supp_62_4_e02007-17__index.html]

Genetic Basis of Emerging Vancomycin, Linezolid, and Daptomycin Heteroresistance in a Case of Persistent Enterococcus faecium Bacteremia — Supplemental material 

# Genetic Basis of Emerging Vancomycin, Linezolid, and Daptomycin Heteroresistance in a Case of Persistent Enterococcus faecium Bacteremia

## Supplemental material

- Supplemental file 1 -

  Fig. S1 and S2, Text S1, and Tables S1 and S2

  PDF, 2.7M
