# Supplementary material for: Genetic Basis of Emerging Vancomycin, Linezolid, and Daptomycin Heteroresistance in a Case of Persistent Enterococcus faecium Bacteremia
Source: Antimicrob Agents Chemother. 2018 Mar 27;62(4):e02007-17. doi: 10.1128/AAC.02007-17 (PMC5913925; doi:10.1128/AAC.02007-17)
Supplement: Supplemental material [file AAC.02007-17_zac004186987s1.pdf]

Supplementary Data for:

The genetic basis of emerging vancomycin, linezolid, and daptomycin heteroresistance in a case of persistent *Enterococcus faecium* bacteremia

Supplementary Methods

Ethical statement
This study was approved by the Icahn School of Medicine at Mount Sinai (ISMMS) Institutional Review Board, New York, USA (IRB-16-01108).

Patient characteristics and antibiotics usage
In late 2015, a 65 year-old male was admitted to Mount Sinai Hospital (MSH) with a necrotic liver abscess. The patient had a history of recurrent hepatocellular carcinoma (HCC) and had undergone a tumor resection, complicated by bile leak necessitating reoperation and conversion to roux-y hepaticojejunostomy. He subsequently developed HCC recurrence and extension into the inferior vena cava, and underwent intra-arterial yttrium-90 radioembolization chemotherapy, which was complicated by development of liver abscess. Percutaneous drainage was performed and a fully susceptible *E. faecalis* grew from the drainage culture. A blood culture taken at the same time was positive for viridans group streptococci. Following drainage, and interventional radiology, the patient was discharged with 14 days of Ampicillin-sulbactam 875-125 q12h (every 12 hours) PO (per os). The patient was readmitted two weeks later due to sepsis, marking the start of our investigations (study day 0). Presence of *E. faecium* was first detected in the blood on day 9 and intermittently afterwards up to day 90, with additional detections of *Candida glabrata*, *Enterobacter cloacae*, and *Stenotrophomonas maltophilia* (Figure 1, main text). Four stool samples and nine urine samples, collected throughout the hospital stay, showed no growth when tested for MDR gram negative bacteria and bacteriuria, respectively. Source control of the liver abscess was addressed with placement of multiple drains by interventional radiology. Over the course of the first 51 days, the patient was treated with 1 gram of intravenous cefepime every 12 hours (IV 1g q12h), ceftazidime (IV 2g q8h), caspofungin (IV 50 mg q12h), daptomycin (IV 350 mg q24h), fluconazole (200mg daily PO), gentamicin (IV 60 mg q8h and 180 mg daily), imipenem (IV 500 mg q6h), levofloxacin (IV 500 mg daily), linezolid (IV 600 mg q12h), pipercillin-tazobactam (PIP-TAZ) (IV 3.375 g q6h), trimethoprim/sulfamethoxazole (TMP-SMX) (1 tablet q12h PO) and vancomycin (IV 1g q12h) (see also Figure 1 in the main text for treatment interval details).

The patient was discharged to home on day 51 with a Peripherally Inserted Central Catheter (PICC) for cefepime (IV 1 g q12h), as well as TMP/SMX (1 tablet q12h PO), fluconazole (200 mg daily PO), and linezolid (600 mg q12h x 12 days PO).

Approximately one month later (day 75), the patient was again readmitted to MSH for sepsis with positive blood cultures for *E. faecium*. At this time the patient was prescribed cefepime (IV 2 g q12h), caspofungin (IV 50 mg q12h), daptomycin (IV 300 mg q24h), imipenem (IV 500mg x1), linezolid (IV 600 mg q12h), and vancomycin (IV 1 g x1). Despite aggressive antibiotic and antifungal treatment, the patient continued to decline and was eventually transferred to palliative care.

Bacterial Isolates, Species Identification and Antibiotic Susceptibility Testing

Bacterial isolates were recovered by sampling from the original blood cultures in the Mount Sinai Hospital Clinical Microbiology Laboratory (CML), and stored in tryptic soy broth with 15% glycerol at -80°C. VITEK 2 (bioMérieux) automated broth microdilution antibiotic susceptibility profiles were obtained for each isolate in the CML according to Clinical and Laboratory Standards Institute (CLSI) 2015 guidelines and reported according to CLSI guidelines (1). Species confirmation was performed with MALDI-TOF (Bruker Biotyper, Bruker Daltonics). For further processing, all isolates were cultured on tryptic soy agar (TSA) plates with 5% sheep blood (blood agar) (ThermoFisher Scientific) under nonselective conditions. Minimum inhibitory concentrations (MIC) were determined in duplicate for vancomycin and linezolid using E-tests (bioMérieux) performed on Mueller Hinton Agar plates (Sigma-Aldrich) and a 0.5 McFarland standard, and for daptomycin using Sensititre GPN3F susceptibility plates (ThermoFisher Scientific) and a cation-adjusted Mueller-Hinton broth.

DNA Preparation and sequencing

Seven strains derived from single colonies of six isolates were selected for sequencing based on their susceptibility profiles. For the hetero-resistant patient isolate *E*, four single colonies were selected and grown separately on Blood Agar plates under nonselective conditions. E-tests were then performed, and a susceptible and a resistant strain were selected for sequencing. All seven strains were grown overnight on Blood Agar plates, followed by DNA extraction using the Qiagen DNeasy Blood & Tissue Kit (Qiagen, 69504) according to the manufacturer's instructions, with modified lysis conditions as follows. Bacterial cells were lysed by suspending cells in 3 μL of 100mg/ml RNase A (Ambion, AM2286) and 10 μL of 100mg/ml lysozyme (Sigma, L1667-1G) for 30 minutes at 37°C, followed by incubation with Proteinase K for one hour at 56°C and two rounds of bead beating of 1 min each using 0.1mm silica beads (MP Bio).

Quality control, DNA quantification, and gDNA library preparation for single molecule real-time (SMRT) sequencing was performed as previously described (2). Briefly, DNA was gently sheared into ~20,000 bp fragments using Covaris G-tube spin columns, and end-repaired before ligating SMRTbell adapters (Pacific Biosciences). The resulting library was treated with an exonuclease cocktail to remove un-ligated DNA fragments, followed by two additional (Ampure XP) purifications steps and Sage Science BluePippin size selection to deplete SMRTbells < 7,000 bp. Libraries were then sequenced on the Pacific Biosciences (PacBio) RS II platform using the P5-C3 sequencing enzyme and chemistry.

Illumina sequencing was performed for assembly finishing. Genomic DNA (1 μg) was sheared to an average fragment size of 200 bp using a Bioruptor Pico sonicator (Diagenode). Amplicon sequence libraries were prepared using the end repair, A-tailing, and adaptor ligation NEBNext DNA library prep modules for Illumina from New England Biolabs, according to the manufacturer's protocol. Following final purification with Ampure XP beads and secondary PCR (8 cycles) to introduce barcoded primers, multiplexed libraries were sequenced on the Illumina HiSeq 2500 platform in a single-end 100 nt-run format to >30x genomic coverage.

Complete genome assembly and finishing

PacBio sequencing data were assembled using HGAP3 version 2.2.0, (3). Illumina reads were then mapped to the curated PacBio assemblies and consensus calling was performed using the mpileup function of samtools (4) to correct errors in the PacBio assembly, which mainly consisted of insertion/deletion errors in homopolymer regions (**Table S1**). Genome circularization, curation and annotation were performed using a custom post-assembly pipeline (https://github.com/powerpak/pathogendb-pipeline) (2). Replicon and origin of replication were identified with PlasmidFinder 1.3 (5) and insertion elements were mapped using ISfinder (6).

Pairwise Genome Comparisons and Phylogenetic Analyses

Multi-locus sequence types (MLST) of each strain genome were determined using the E. *faecium* PubMLST database (http://pubmlst.org/efaecium/). Single nucleotide and structural variants between strains were identified by comparing each genome to the first isolate strain using NUCmer (Version 3.1) (7). Nonsynonymous sequence changes in predicted ORFs were confirmed by multiple sequence alignments using MUSCLE (8).

For phylogenetic analyses, 761 complete genome, chromosome, scaffold and contig *E. faecium* sequences were downloaded from GenBank on 1 July, 2017. Phylogenetic analysis of all ST736 genomes was performed using Parsnp, part of the Harvest bioinformatics tool suite (Version 1.1.2), using default MUMi (9) <0.01 cutoff settings and filtering SNPs located in regions of recombination (10). For plasmid recombination analyses, shared short homologous regions were identified by BLAST+(11) and Contiguity (12).

Antibiotic Resistance Gene Annotation

Antibiotic resistance gene and variants were annotated by comparing to a manually curated database of known *E. faecium* resistance determinants from literature. BLASTP was used to identify the presence of genes in each strain genome, with sequence identity cutoff ≥90% and an e-value cut-off ≤ 1e-10. Resistance variants were identified by BLASTP alignment relative to the wild-type gene protein sequence. Only exact matches to variants identified in literature were considered. An analogous approach with BLASTN was used to identify 23S rRNA variants on nucleotide sequences.

Accession numbers

Genome sequences have been deposited in Genbank, Bioproject accession number PRJNA407447 (http://www.ncbi.nlm.nih.gov/bioproject/407447).

Supplementary Tables

Table S1. Genome assembly statistics.

Strain	Sequence	Identifier	Replicon	Size (bp)	# SNVs[a)]	# SVs[b)]	PacBio[c)]	Illumina[d)]	Corrections[e)]
A	Chromosome	MSHS ER03933.3A	*repUS12*	2,863,296	-	-	89.9	1134.2	8
	Plasmid 1	pER03933.3A.1	*repUS15*	199,876	-	-	171.0	2079.4	2
	Plasmid 3	pER03933.3A.3	*rep18*	10,647	-	-	28.3	5328.8	0
	Plasmid 4	pER03933.3A.4	Unknown	4,374	-	-	0.6	7714.8	0
B	Chromosome	MSHS ER04120.3A	*repUS12*	2,869,275	2	6	83.3	704.4	10
	Plasmid 1	pER04120.3A.1	*repUS15*	199,876	-	-	173.1	1415.4	0
	Plasmid 2	pER04120.3A.2	*rep17*	40,911	-	-	121.5	1299.9	0
	Plasmid 3	pER04120.3A.3	*rep18*	10,647	-	-	23.1	4719.4	0
	Plasmid 4	pER04120.3A.4	Unknown	4,374	-	-	0.80	6169.8	0
C	Chromosome	MSHS ER04462.3A	*repUS12*	2,867,770	4	5	97.7	598.2	151
	Plasmid 1	pER04462.3A.1	*repUS15*	199,068	-	1	182.1	1086.6	0
	Plasmid 2	pER04462.3A.2	*rep17*	40,911	-	-	132.1	985.4	0
	Plasmid 3	pER04462.3A.3	*rep18*	10,647	-	-	27.8	3098.5	0
	Plasmid 4	pER04462.3A.4	Unknown	4,374	-	-	0.5	4733.5	0
D	Chromosome	MSHS ER04484.3A	*repUS12*	2,869,064	6	5	101.1	1024.4	126
	Plasmid 1	pER04484.3A.1	*repUS15*	202,553	1	2	236.2	2145.5	1
	Plasmid 2	pER04484.3A.2	*rep17*	40,911	-	-	150.5	1812.1	0
	Plasmid 3	pER04484.3A.3	*rep18*	10,647	-	-	34.6	4769.3	2
	Plasmid 4	pER04484.3A.4	Unknown	4,375	1	-	4.7	8171.0	4
E-VS	Chromosome	MSHS ER04562.3A	*repUS12*	2,867,667	7	6	93.4	1008.8	28
	Plasmid 1	pER04562.3A.1	*repUS15*	202,540	1	2	289.7	2754.9	4
	Plasmid 3	pER04562.3A.3	*rep18*	10,647	-	-	136.7	1871.2	0
	Plasmid 4	pER04562.3A.4	Unknown	4,374	2	-	77.7	3846.1	2
	Plasmid 5	pER04562.3A.5	*rep17*	81,514	-	-	1.3	7407.0	0
E-VR	Chromosome	MSHS ER04526.5A	*repUS12*	2,870,954	5	5	125.5	55.5	6
	Plasmid 1	pER04526.5A.1	*repUS15*	202,540	1	2	273.6	117.3	3
	Plasmid 2	pER04526.5A.2	*rep17*	40,911	-	-	223.1	115.0	0
	Plasmid 3	pER04526.5A.3	*rep18*	10,647	-	-	88.0	218.9	0
	Plasmid 4	pER04526.5A.4	Unknown	4,374	-	-	2.3	127.1	8
F	Chromosome	MSHS ER04619.3A	*repUS12*	2,870,397	7	6	84.0	1067.4	32
	Plasmid 1	pER04619.3A.1	*repUS15*	201,207	1	1	176.2	2165.9	4
	Plasmid 3	pER04619.3A.3	*rep18*	10,647	-	-	31.3	4635.3	0
	Plasmid 4	pER04619.3A.4	Unknown	4,374	-	-	0.4	8671.1	0

Footnotes
a) # of single nucleotide variants (SNV) relative to reference strain *A*. For Plasmid 2, # of SNVs are relative to strain *B*.
b) # of structural variants (SV) relative to reference strain *A*. For Plasmid 2, # of SVs are relative to strain *B*.
c) PacBio Coverage
d) Illumina Coverage
e) # of PacBio bases corrected with illumina sequencing data

Table S2. Summary of genomic features and variants compared to reference strain *A*

Event type[a)]	ID[b)]	Gene	Description of gene product	Start[c)]	Reference Strain A[d)]	Strain B[e)]	Strain C[e)]	Strain D[e)]	Strain E-VS[e)]	Strain E-VR[e)]	Strain F[e)]	Notes[f)]
**Chromosome**												
Frameshift insertion + duplication	00030		Hypothetical Protein	34,070		1,463						IS16, family IS256
Nonsynonymous SNV	00279		Hypothetical Protein	288,008	C	A						
Intergenic SNV	-		-	380,976	G		T					
Frameshift deletion	00395	*helD_1*	Helicase IV	411,408	A				-			
Frameshift insertion + duplication	-		-	531,339							1,324	IS256, family IS256
Non-frameshift insertion + duplication	-		-	710,338				1,323				IS256, family IS256
Nonsynonymous SNV	00941	*fabF*	3-oxoacyl-[acyl-carrier-protein] synthase 2	970,332	G			A	A	A	A	
Frameshift insertion + duplication	01354		Putative frv operon regulatory protein	1,404,686		1,678	1,678	1,678	1,678	1,678	1,678	IS1678, family IS1380
Frameshift deletion	01494	*licR_2*	Putative licABCH operon regulator	1,535,231	T		-					
Frameshift insertion	01504		Hypothetical Protein	1,548,214							G	
Frameshift deletion	01572		Transposase	1,622,364	C				-			
Synonymous SNV	01697		Phage portal protein	1,753,662	A		G					
Recombination	01711<br>01721<br>01732		Hypothetical Protein<br>Hypothetical Protein<br>Hypothetical Protein	1,761,042			91					
Frameshift insertion + duplication	-		-	1,811,321		1,466						IS16, family IS256
Frameshift insertion + duplication	-		-	1,844,688		1,324	1,324	1,324	1,324		1,324	ISEfm2, family IS256
Large intergenic substitution	-		-	1,844,689						61		
Frameshift insertion	-		-	1,844,749						1,268		ISEfm2, family IS256
Non-Frameshift insertion + duplication	01870		Hypothetical Protein	1,900,489		1,338						ISEf1, family IS256
Nonsynonymous SNV	01877	*epsL*	Putative sugar transferase EpsL	1,908,259	A		T					
Non-Frameshift insertion + duplication	-		23S rRNA	2,168,664				1,416	1,416	1,416	1,416	ISEnfa3, family IS3
Nonsynonymous SNV	02218	*dppE*	Dipeptide-binding protein DppE precursor	2,238,674	C			T			T	
Frameshift insertion + duplication	-		-	2,317,576					1,325			ISEfm2, family IS256
rRNA SNV	-		23S rRNA	2,453,917	G				T			
rRNA SNV	-		23S rRNA	2,564,745	G			T	T	T	T	
Frameshift insertion	-		-	2,671,815							1	
Non-frameshift deletion	02667		Transposase, mutator family	2,727,302	1323	-						IS256, family IS256
Reshuffling	-		-	2,727,302			1,323	1,323	1,323	1,323	*1,323*	IS256, family IS256, deleted and reinserted at alternate location. Inverted copy in isolate F
Frameshift insertion +	02676	*znuA*	High-affinity zinc uptake system binding-	2,736,728					1,889			ISEfa4, family IS200/IS605

duplication			protein ZnuA precursor									
Frameshift insertion + duplication	02686	*ykoD*	Putative HMP/thiamine import ATP-binding protein YkoD	2,745,430			1,465					IS16, family IS256
Stop-gain	02706		Hypothetical Protein	2,764,659	A	T		T	T	T	T	
Frameshift insertion	02728		Hypothetical Protein	2,783,706				1	1	1		
Frameshift insertion + duplication	02728		Hypothetical Protein	2,783,706							1,324	IS256, family IS256
rRNA SNV	-		23S rRNA	2,840,480	G			T	T	T	T	
**Plasmid 1**												
Frameshift deletion	02864		Hypothetical Protein	62,323	A			-			-	
Frameshift deletion	02864		Hypothetical Protein	63,604	T				-			
Non-Frameshift insertion + duplication	02877		Hypothetical Protein	71,585				1338				ISEf1, family IS256
Non-Frameshift insertion + duplication	-		-	91,534				1323	1323	1323	1323	IS256, family IS256
Nonsynonymous SNV	02929		Integrase core domain protein	122,388	C					T		
Frameshift insertion + duplication	-		-	123,139						1324		IS256, family IS256
Frameshift insertion + duplication	-		-	123,431					1337			ISEf1, family IS256
Frameshift deletion	00156		Hypothetical Protein	152,252	808		-					
Frameshift deletion	02864		Hypothetical Protein	62,323	A			-			-	
Frameshift deletion	02864		Hypothetical Protein	63,604	T				-			
**Plasmid 4**												
Nonsynonymous SNV	03026		Hypothetical Protein	1,380	G				C			
Nonsynonymous SNV	03029		Plasmid recombination enzyme	2,805	A				C			
Intergenic SNV	-		-	4,230				T				

Footnotes

a. SNV - Single nucleotide variant.
b. PROKKA gene ID in reference strain *A*.
c. Start coordinate of the event in reference strain *A*.
d. Reference base (for SNVs) or size of insertion sequence (for deletion events) in reference strain *A*.
e. Nucleotide changes (for SNVs) or size of the structural changes compared to reference strain *A*.
f. IS - Insertion sequence

Supplementary Figures

Figure S1. Maximum likelihood phylogenetic tree based on core genome SNVs for case strain genomes and 34 draft or complete ST736 *E. faecium* genomes present in Genbank. The 34 ST736 genomes had a calculated 75% shared core genome. SNV distances are indicated relative to the first patient strain *A*.

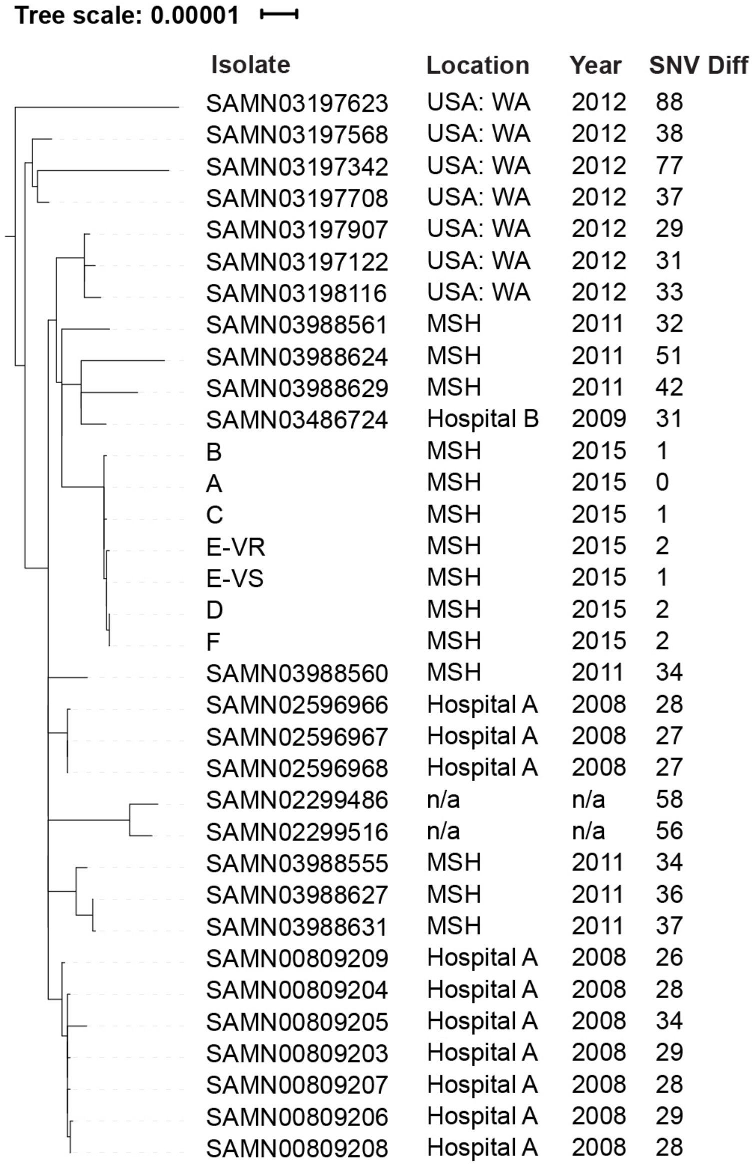

Isolate	Location	Year	SNV Diff
SAMN03197623	USA: WA	2012	88
SAMN03197568	USA: WA	2012	38
SAMN03197342	USA: WA	2012	77
SAMN03197708	USA: WA	2012	37
SAMN03197907	USA: WA	2012	29
SAMN03197122	USA: WA	2012	31
SAMN03198116	USA: WA	2012	33
SAMN03988561	MSH	2011	32
SAMN03988624	MSH	2011	51
SAMN03988629	MSH	2011	42
SAMN03486724	Hospital B	2009	31
B	MSH	2015	1
A	MSH	2015	0
C	MSH	2015	1
E-VR	MSH	2015	2
E-VS	MSH	2015	1
D	MSH	2015	2
F	MSH	2015	2
SAMN03988560	MSH	2011	34
SAMN02596966	Hospital A	2008	28
SAMN02596967	Hospital A	2008	27
SAMN02596968	Hospital A	2008	27
SAMN02299486	n/a	n/a	58
SAMN02299516	n/a	n/a	56
SAMN03988555	MSH	2011	34
SAMN03988627	MSH	2011	36
SAMN03988631	MSH	2011	37
SAMN00809209	Hospital A	2008	26
SAMN00809204	Hospital A	2008	28
SAMN00809205	Hospital A	2008	34
SAMN00809203	Hospital A	2008	29
SAMN00809207	Hospital A	2008	28
SAMN00809206	Hospital A	2008	29
SAMN00809208	Hospital A	2008	28

Figure S2. Circos plots of whole-genome alignments for all case strains.

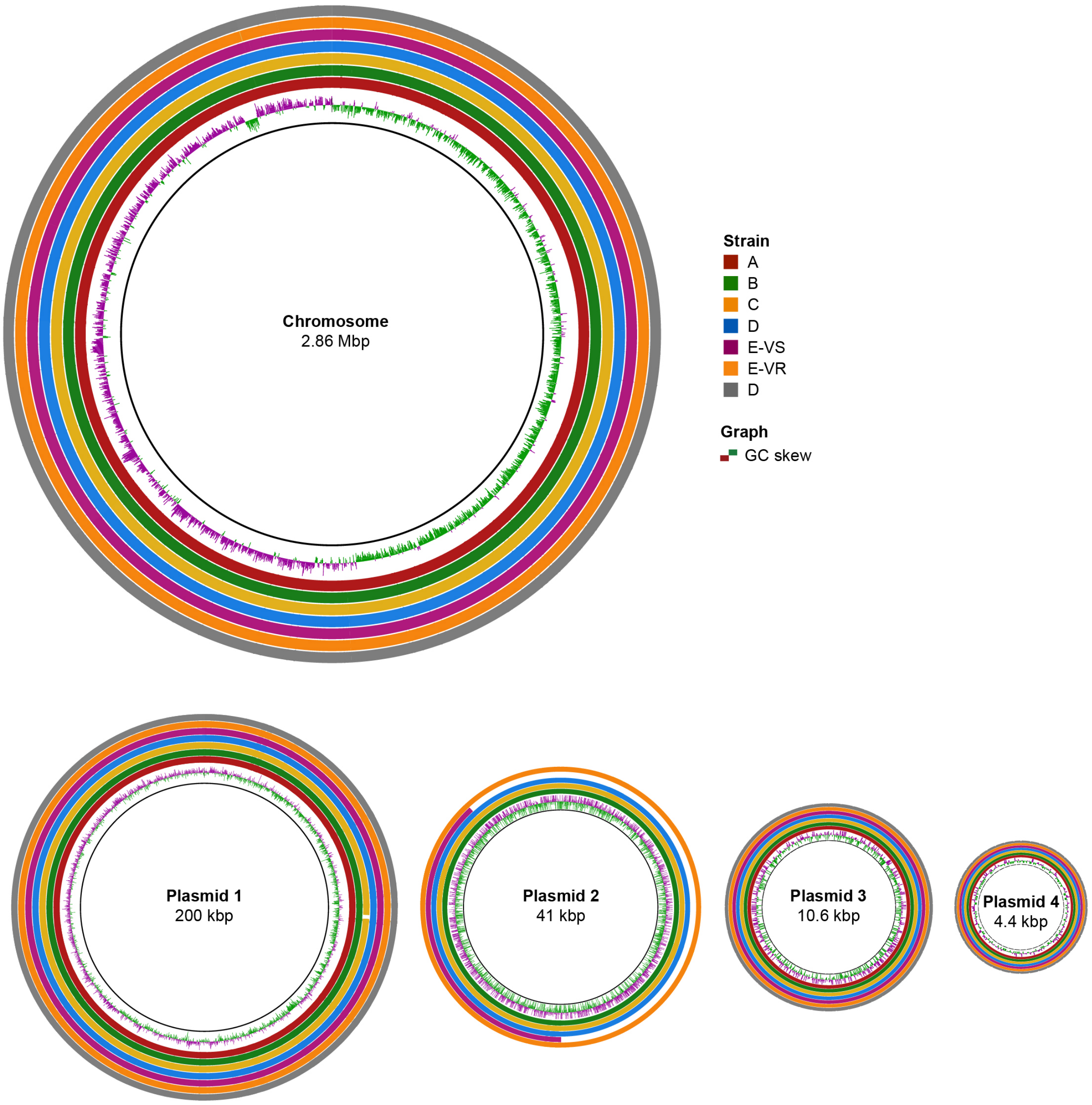

References

1. Wayne PA. 2015. CLSI. Performance Standards for Antimicrobial Susceptibility Testing; Twenty-Fifth Informational Supplement. CLSI Document M100-S25, Clinical and Laboratory Standards Institute.

2. Bashir A, Attie O, Sullivan M, Sebra R, Singh KV, Altman D, Pak T, Dutta J, Chacko K, Webster E, Lewis M, Hamula C, Delli Carpini KW, Murray BE, Kasarskis A, van Bakel H, Huprikar S. 2017. Genomic confirmation of vancomycin-resistant Enterococcus transmission from deceased donor to liver transplant recipient. PLoS One 12:e0170449.

3. Chin CS, Alexander DH, Marks P, Klammer AA, Drake J, Heiner C, Clum A, Copeland A, Huddleston J, Eichler EE, Turner SW, Korlach J. 2013. Nonhybrid, finished microbial genome assemblies from long-read SMRT sequencing data. Nat Methods 10:563–569.

4. Li H, Handsaker B, Wysoker A, Fennell T, Ruan J, Homer N, Marth G, Abecasis G, Durbin R, 1000 Genome Project Data Processing Subgroup. 2009. The Sequence Alignment/Map format and SAMtools. Bioinformatics 25:2078–2079.

5. Carattoli A, Zankari E, Garcìa-Fernandez A, Volby Larsen M, Lund O, Villa L, Aarestrup FM, Hasman H. 2014. PlasmidFinder and pMLST: in silico detection and typing of plasmids. Antimicrob Agents Chemother.

6. Siguier P, Perochon J, Lestrade L, Mahillon J, Chandler M. 2006. ISfinder: the reference centre for bacterial insertion sequences. Nucleic Acids Res 34:D32–6.

7. Delcher AL, Salzberg SL, Phillippy AM. 2003. Using MUMmer to identify similar regions in large sequence sets. Curr Protoc Bioinformatics Chapter 10:Unit 10 3.

8. Edgar RC. 2004. MUSCLE: multiple sequence alignment with high accuracy and high throughput. Nucleic Acids Res 32:1792–1797.

9. Deloger M, El Karoui M, Petit M-A. 2009. A genomic distance based on MUM indicates discontinuity between most bacterial species and genera. J Bacteriol 191:91–99.

10. Treangen TJ, Ondov BD, Koren S, Phillippy AM. 2014. The Harvest suite for rapid core-genome alignment and visualization of thousands of intraspecific microbial genomes. Genome Biol 15:524.

11. Camacho C, Coulouris G, Avagyan V, Ma N, Papadopoulos J, Bealer K, Madden TL. 2009. BLAST+: architecture and applications. BMC Bioinformatics 10:421.

12. Sullivan MJ, Zakour NLB, Forde BM, Stanton-Cook M, Beatson SA. 2015. Contiguity: contig adjacency graph construction and visualisation. PeerJ PrePrints.
